# Linked Entity Attribute Pair (LEAP): A Harmonization Framework for Data Pooling

**DOI:** 10.1200/CCI.20.00037

**Published:** 2020-08-05

**Authors:** Stacy Thomas, Tara Lichtenberg, Kristen Dang, Michael Fitzsimons, Robert L. Grossman, Ritika Kundra, Jessica A. Lavery, Michele L. Lenoue-Newton, Katherine S. Panageas, Charles Sawyers, Nikolaus D. Schultz, Sahussapont J. Sirintrapun, Umit Topaloglu, Angelica Welch, Thomas Yu, Ahmet Zehir, Stuart Gardos

**Affiliations:** ^1^ Memorial Sloan Kettering Cancer Center, New York, NY; ^2^ Center for Translational Data Science, University of Chicago, Chicago, IL; ^3^ Sage Bionetworks, Seattle, WA; ^4^ University of Illinois at Chicago, Chicago, IL; ^5^ Department of Epidemiology and Biostatistics, Memorial Sloan Kettering Cancer Center, New York, NY; ^6^ Vanderbilt-Ingram Cancer Center, Vanderbilt University Medical Center, Nashville, TN; ^7^ Human Oncology and Pathogenesis Program, Memorial Sloan Kettering Cancer Center, New York, NY; ^8^ Department of Pathology, Memorial Sloan Kettering Cancer Center, New York, NY; ^9^ Cancer Biology, Wake Forest University School of Medicine, Winston Salem, NC; ^10^ Information Systems, Memorial Sloan Kettering Cancer Center, New York, NY

## Abstract

**PURPOSE:**

As data-sharing projects become increasingly frequent, so does the need to map data elements between multiple classification systems. A generic, robust, shareable architecture will result in increased efficiency and transparency of the mapping process, while upholding the integrity of the data.

**MATERIALS AND METHODS:**

The American Association for Cancer Research’s Genomics Evidence Neoplasia Information Exchange (GENIE) collects clinical and genomic data for precision cancer medicine. As part of its commitment to open science, GENIE has partnered with the National Cancer Institute’s Genomic Data Commons (GDC) as a secondary repository. After initial efforts to submit data from GENIE to GDC failed, we realized the need for a solution to allow for the iterative mapping of data elements between dynamic classification systems. We developed the Linked Entity Attribute Pair (LEAP) database framework to store and manage the term mappings used to submit data from GENIE to GDC.

**RESULTS:**

After creating and populating the LEAP framework, we identified 195 mappings from GENIE to GDC requiring remediation and observed a 28% reduction in effort to resolve these issues, as well as a reduction in inadvertent errors. These results led to a decrease in the time to map between OncoTree, the cancer type ontology used by GENIE, and International Classification of Disease for Oncology, 3rd Edition, used by GDC, from several months to less than 1 week.

**CONCLUSION:**

The LEAP framework provides a streamlined mapping process among various classification systems and allows for reusability so that efforts to create or adjust mappings are straightforward. The ability of the framework to track changes over time streamlines the process to map data elements across various dynamic classification systems.

## INTRODUCTION

Advances in molecular biology continue to expand disease-relevant data faster than existing classification systems can incorporate this information. In 2011, a report from the US National Research Council called for a new taxonomy in pursuit of “precision medicine,” a phrase to which the report gave prominence. The council, funded by a grant from the National Academy of Sciences and the National Institutes of Health, envisioned that this taxonomy would be developed iteratively over decades, incorporating input from contributors with various disease specialties using shared data. While emphasizing the importance of data-sharing efforts, they also outlined institutional, cultural, and regulatory barriers to broad data-sharing initiatives.^1^ These issues included, but were not limited to, a lack of incentive for researchers to share data for projects requiring high effort. Data-sharing challenges persisted 5 years later. In 2016, the Blue Ribbon Panel of scientific experts (part of then–Vice President Joe Biden’s Cancer Moonshot Initiative) echoed the barriers listed in the council report and added that researchers frequently lack the “expertise and support structure” to conform their data to standards to make them sharable.^2^

**CONTEXT**

**Key Objective**
The harmonization of classification systems is frequently ad hoc and infrequently transparent. A robust architecture to store and share mappings is necessary to reduce effort and error, and to increase transparency.
**Knowledge Generated**
After implementing the Linked Entity Attribute Pair (LEAP) framework, we identified 195 mapping errors and reduced remediation effort by 28%. Using LEAP reduced our mapping time from months to less than 1 week.
**Relevance**
The advance of precision medicine relies heavily on data sharing and analysis, yet there is a lack of widely accepted standard terminology that results in different classification systems being used for annotation. The LEAP framework streamlines and reduces errors in the mapping process, while creating transparency for data submitters and consumers to facilitate reproducibility.

In 2016, the National Cancer Institute (NCI) launched the Genomic Data Commons (GDC) as a platform for sharing data across international cancer genomic projects.^3^ Since then, several large programs have deposited genomics data into GDC, including The Cancer Genome Atlas, Foundation Medicine, and the TARGET (Therapeutically Applicable Research to Generate Effective Treatments) initiative.

In response to the Cancer Moonshot Initiative, the American Association for Cancer Research commenced the Genomics Evidence Neoplasia Information Exchange (GENIE) project. GENIE collects de-identified clinical and genomic data from tens of thousands of patients treated at various international institutions—beginning with 8 founding centers and expanding to current total of 18—to further research for precision cancer medicine. GENIE has a partnership agreement with Sage Bionetworks to facilitate data sharing, versioning, and provenance.

As part of its commitment to open science and the democratization of genomic data, GENIE has partnered with GDC as a secondary repository. Furthermore, this partnership would also lay the groundwork for recognition of GENIE as a Food and Drug Administration–designated public genetic variants repository. We—collaborators from Memorial Sloan Kettering Cancer Center (MSK) and Sage Bionetworks, in partnership with GENIE—describe our efforts for data submission to the GDC. The initial effort to submit data from GENIE to GDC was unsuccessful because of mapping inconsistencies between the different diagnostic classification systems used by each platform, which we expand on in this article. Furthermore, initial efforts to resolve the inconsistencies were greatly hindered by our use of a spreadsheet to manage the mappings, which created confusion and did not enforce any logic between dependent elements.

In the subsequent sections, we describe the requirements and final approach used to manage the ongoing process of mapping between the classification systems used by GENIE and GDC. In addition we outline this structured process and the Linked Entity Attribute Pair (LEAP) framework used to store the mappings, and share the scripts to recreate the solution, with the intention of serving as a resource for other initiatives that need to store customized, versioned mappings of data elements between classification systems.^4^ The LEAP framework has been used at MSK to link other classification systems as well, allowing for streamlined, updated groupings within our Enterprise Data Warehouse (EDW). By bridging these data within back-end systems and then feeding the data to the EDW, physicians and researchers can easily explore harmonized data—with the original data still easily accessible—using the terminology of their choice. This approach reduces the time to insight by removing the burden of data harmonization from the investigators.

## MATERIALS AND METHODS

### Components and Classifications of Cancer Diagnosis

The multidimensional nature of the cancer diagnosis process starts with a pathologic diagnosis, which is usually documented in a semistructured/unstructured pathology report. Despite the College of American Pathologists’ synoptic reporting efforts, pathologists still use nonstandard descriptions for final diagnosis. A cancer diagnosis is composed of 2 different elements, the first being the originating site of the tumor (ie, topographic), such as left ovary. The second element is the morphology of the tumor, such as seromucinous carcinoma. Clinically and operationally, either the site alone is used to describe the cancer (ie, ovarian cancer) or the site and morphology are combined (ie, ovarian seromucinous carcinoma). Table 1 details the ways that various classification systems and specific versions of these systems would classify seromucinous carcinoma found in the left ovary.

**TABLE 1.**
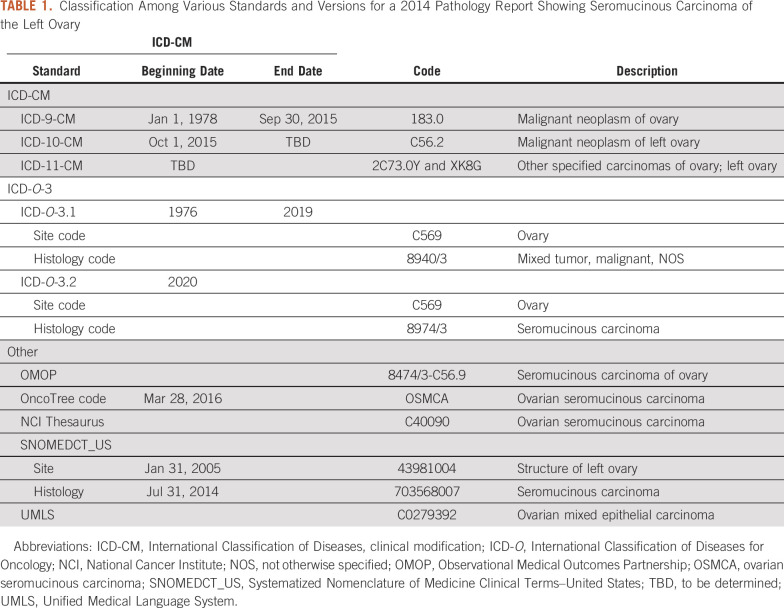
Classification Among Various Standards and Versions for a 2014 Pathology Report Showing Seromucinous Carcinoma of the Left Ovary

The International Classification of Diseases (ICD) is a foundation from the World Health Organization that maintains standards for the identification and reporting of diseases.^5^ The classification system used to document all diagnoses for billing and reimbursement are the clinical modification (CM) codes. For cancer diagnoses, the current version (10th revision; ICD-10-CM) only describes the site of the tumor. The next version (ICD-11-CM) will incorporate morphology into the classification, but an adoption date in the United States has not been finalized. Separately, International Classification of Diseases for Oncology (ICD-*O*) provides a classification system specifically for oncology diagnoses. The current version, ICD-*O*-3, splits cancer diagnoses into site and morphology. ICD-*O* coding is used for cancer reporting within tumor registries. The GDC uses an ICD-*O*-3–based classification system to describe tumors.

Due in part to GENIE’s goal to further research into rare cancers, OncoTree was chosen as the classification system for the histopathologic diagnosis of samples.^6^ OncoTree was developed at MSK to allow diagnostic molecular pathologists to classify cancer diagnoses from pathology reports associated with tumor samples sent for genomic testing. It is organized by the site of disease, using the fields tissue (for site), main type, and name to describe diagnoses. It also provides an abbreviated code for documentation. OncoTree is updated approximately every 6 weeks based on ongoing feedback from diagnostic molecular pathologists to the OncoTree Committee, who manage additions and modifications to OncoTree (OncoTree versions can be viewed via http://OncoTree.mskcc.org/). A new version of OncoTree is adopted by GENIE annually. The detailed files from the 2 OncoTree versions used thus far in the releases GENIE has submitted to GDC are available in the Data Supplement.

### Limitations With Initial Approach to Mapping From OncoTree to GDC Standards

GENIE is the largest consortium contributing to the GDC with its first submission—released in the fourth quarter of 2019—containing 44,756 cases.^7^ Unlike other data-sharing programs that have previously submitted data to GDC, there are no requirements for GENIE that predetermine the histologic subtypes or sites of disease that will be included. Although the broad inclusivity of GENIE is beneficial for downstream researchers looking for a variety of subtypes, it is challenging to ensure correct classification and harmonization in a way that is meaningful to others trying to access the data. This is particularly true when the data are contributing to efforts that use a different classification system, like GDC.

As previously mentioned, when GENIE initially began work for its first submission to GDC, a spreadsheet for mapping diagnostic terms was created and exchanged between GENIE and GDC. In the spreadsheet, each OncoTree code and sample type—primary, metastasis, and unspecified—were individually mapped to the 7 data elements GDC required for diagnosis (primary diagnosis, tissue or organ of origin, tumor grade, morphology, site of resection or biopsy, primary site, and disease type). Individually mapping these data elements resulted in a sheet with 858 rows and 13 columns, which is included in the Data Supplement. Because of the denormalized structure of the spreadsheet, errors were introduced during the mapping process that resulted in the failure of the initial submission attempt. These errors, shown in Table 2, included but were not limited to terminology not listed in GDC’s data dictionary (n = 74, of which 99% were due to differences in capitalization), mismatched values for related fields (n = 53), and inaccurate mappings to both tissue_or_organ_of_origin and morphology (n = 96). It became clear that a data model was needed to address several needs, such as the easy viewing of relationships and the ability to update mappings while ensuring strong data integrity and semantic meaning. The hybrid Entity–Attribute–Value (EAV) model offered all these advantages and led us to create the LEAP framework based on the design of the model.

**TABLE 2.**
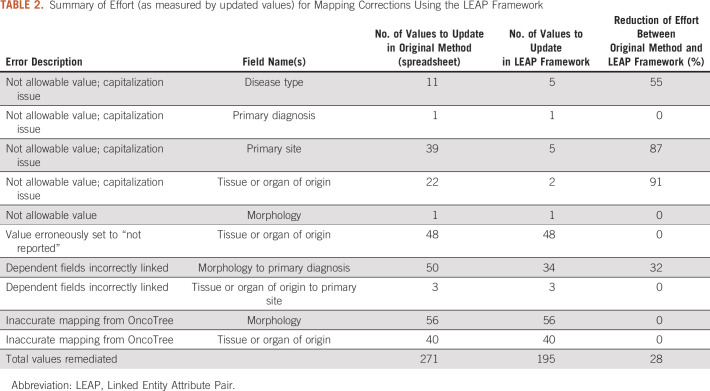
Summary of Effort (as measured by updated values) for Mapping Corrections Using the LEAP Framework

### Overview of Hybrid EAV Model

The EAV model has been widely used to classify clinical data for many years, for example, in clinical data repositories such as Cerner and 3M, as well as in clinical study data management systems like TrialDB.^8^ The EAV model allows systems to capture heterogenous data across attributes. For instance, rather than having separate columns for each field in a questionnaire with a yes/no answer, the EAV model allows the data to be stored as rows, with both the field name and the field value as data elements populated only for those records for which the information is applicable. Typically, EAV models can prove unwieldy with dense data, so we were challenged to design a better EAV model that reduces the technical expertise required to manage the data.^9^ The Hybrid EAV design proposed by Peter Larsson^10^—on which our model is based—strives to make the EAV model more flexible, versatile, smaller, and faster than traditional EAV models.

### Hybrid EAV and LEAP

**FIG 1.**
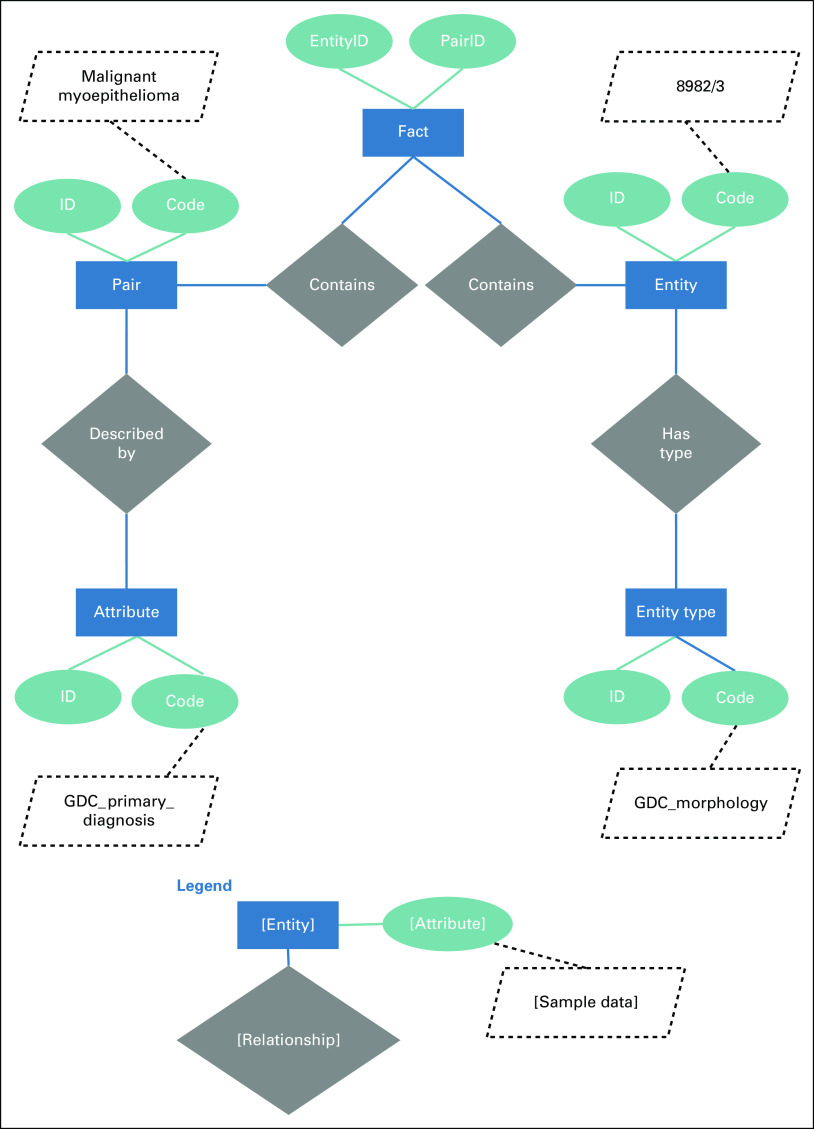
Diagram using Chen’s Notation of an entity within Linked Entity Attribute Pair (LEAP) framework. GDC, Genomic Data Commons; ID, identification.

Larsson’s Hybrid EAV model moves the EAV model into a relational database format by storing the entities, attributes, and values (called “pairs”) in separate tables and adds an entity type table. The model then uses a dimensional structure by joining the tables by their unique numeric identifiers through a fact table. A notable difference between Larsson’s Hybrid EAV model and LEAP is that Larsson’s model includes a table to store database statistics to optimize querying large volumes of data. This is due to its significantly larger database of 1.2 petabytes, compared with LEAP’s 15 megabytes. Another minor change is that we removed the EntityTypeID from the fact table, because it is already referenced in the entity table. Figure 1 displays the direct relationships of objects in our Hybrid EAV model, and a complete Entity-Relationship (ER) diagram is included in the Data Supplement.

Referential integrity, triggers for building the history tables, and a highly dimensional structure were introduced to create a robust system that would be agile enough to incorporate changes in various classification systems and data domains. Importantly, this system needed to be stable enough to present the relationships in a variety of ways while maintaining a detailed history of updates.

**FIG 2.**
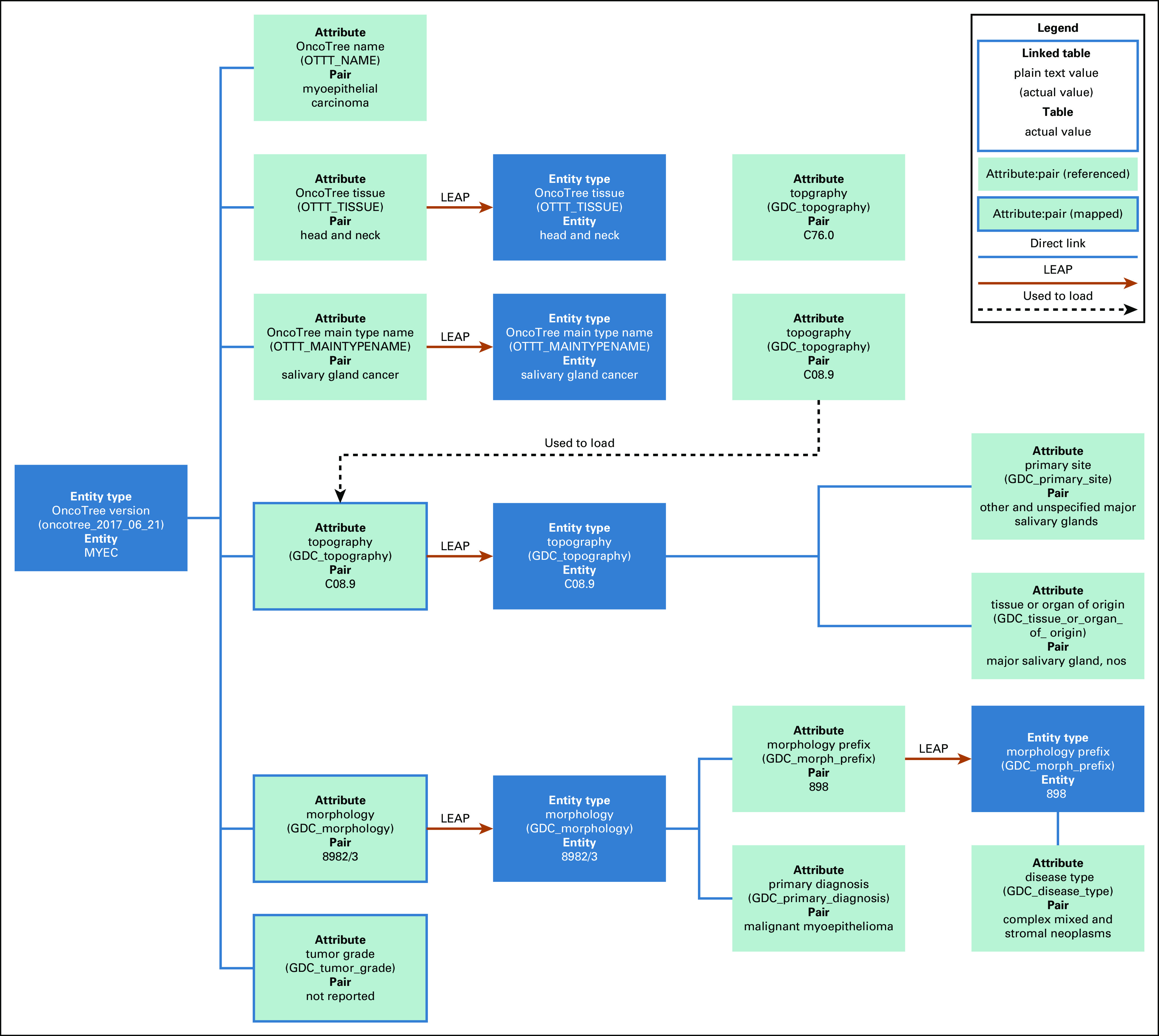
Example of Genomics Evidence Neoplasia Information Exchange (GENIE) to Genomic Data Commons (GDC) mapping in Linked Entity Attribute Pair (LEAP) framework. MYEC, myoepithelial carcinoma of salivary gland origin; OTTT, OncoTree tumor type; UMLS, Unified Medical Language System.

Once the tables were created, we loaded entities and attributes with existing relationships, shown in Figure 2, with attribute:pair (referenced) objects. These included GDC entities such as topography and morphology with their related attributes, as well as OncoTree codes and their related attributes, as shown in Figure 2, via the direct links. The key differentiator of LEAP from Larsson’s Hybrid EAV model is that LEAP allows entities to inherit attributes from other entities, which is represented by the LEAP arrows in Figure 2. For example, in Figure 2, OncoTree code MYEC (myoepithelial carcinoma of salivary gland origin) links to all morphology information through the GDC morphology:8982/3 mapped attribute:pair combination. The GDC describes this morphology as malignant myoepithelioma, and MYEC maps to this description through the link to GDC morphology:8982/3. This separate, yet linked, aspect of LEAP allows users to connect dynamic classification systems, while isolating specific data elements to reduce the risk of errors when updating. An example of this is the OncoTree code STMYEC (soft tissue myoepithelial carcinoma) that is also mapped to GDC morphology:8982/3. If GDC changed the primary diagnosis description for 8982/3 from malignant myoepithelioma to “myoepithelioma, malignant,” LEAP requires only 1 update, which is to the primary diagnosis pair information for GDC morphology:8982/3. This 1 update would cascade to both MYEC and STMYEC through the link to GDC morphology. Allowing targeted updates reduces the risk of both inadvertent errors and data degradation.

### Storing LEAP

Because Sage Bionetworks handles both the data harmonization of submissions to GENIE from various centers and the submission of the aggregated data from GENIE version releases to GDC, we explored the option of using their data-sharing platform, Synapse, to hold the tables for the Hybrid EAV model.^11^ However, we discovered that tables in Synapse do not have the ability to enforce referential integrity, which is key for the Hybrid EAV model to be successful. In addition joining tables in Synapse to query the data is computationally inefficient. Therefore, we stored the LEAP framework at MSK using Structured Query Language (SQL) Server 2017 and supplied information from the model to relational Synapse tables for Sage Bionetwork for submission of GENIE data to GDC. An ER diagram of the relational Synapse tables, as well as the data used to populate the tables, is included in the Data Supplement.

## RESULTS

### Streamlining Remediation

**FIG 3.**
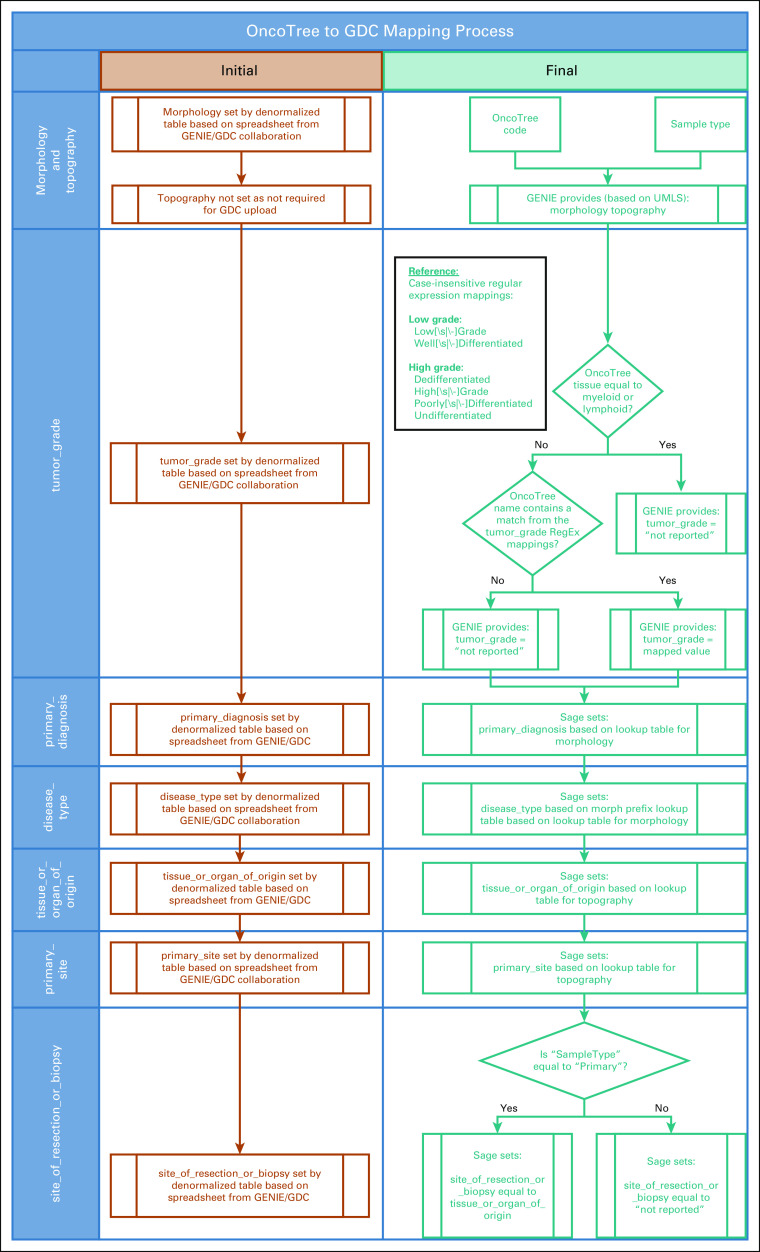
Overview of Genomics Evidence Neoplasia Information Exchange (GENIE) to Genomic Data Commons (GDC) mapping process (both initial and final). UMLS, Unified Medical Language System.

Before implementing the LEAP framework, we were only able to identify errors individually, because submissions to the GDC through their application programming interface failed. In addition we had no way of systematically reviewing mapping patterns for grouping errors. After implementing the LEAP framework, a total of 195 term mappings requiring remediation were immediately identified. The new approach allowed us to reduce our remediation time for allowable value and errors related to dependent fields by 28% (Fig 3; summarized in Table 2, with detailed data available in the Data Supplement).

### Subsequent Mapping of OncoTree to ICD-*O*-3 Standards

The effort to map the first version of OncoTree used by GENIE (oncotree_2017_06_21) took several months. Using the LEAP framework, the next version of OncoTree (oncotree_2018_06_01) was mapped in less than 40 hours. That version had 146 new OncoTree nodes used in the GENIE 6.1 release. Because oncotree_2018_06_01 incorporated detailed feedback on expanding the hematologic cancers, the greatest changes were from the OncoTree codes with blood and lymph OncoTree tissue, which were adapted and expanded into myeloid and lymphoid tissues respectively. As such, the greatest number of additions were to myeloid (75; 51%) and lymphoid (30; 20%).

## DISCUSSION

Data element mapping is frequently conducted ad hoc, based on specific project needs and generally via spreadsheet.^12^ Although the traditional approach is appealing because it requires little investment in time and effort initially, it can be more costly in the long term because of inadvertent errors, revolving classification standards, and challenges around reproducibility.^4,12^ Conversely, the creation of the LEAP framework and the initial mappings required a considerable amount of time and effort, but the reduction in effort in both mapping and troubleshooting immediately exceeded the initial time expenditure.

We completed our submission process from GENIE to GDC in July 2019. In October, the NCI announced the launch of the Center for Cancer Data and Harmonization, which is planned to take more than 3 years to fully develop. This initiative will focus on projects affiliated with the NCI.^13^ Although this is an important and exciting announcement that may address many issues that cancer data-sharing initiatives have historically faced, we believe that the framework we are proposing in this article has value and merit as a more immediate solution. In the interim, the LEAP framework can be applied within cancer initiatives, and more broadly, it can be applied to data-sharing projects outside the scope of cancer.

At MSK, the LEAP framework is also used for mapping: ICD-9-CM and ICD-10-CM codes to Clinical Classification Software groupings, Current Procedural Terminology (CPT) codes to CPT groupings, and Logical Observation Identifiers Names and Codes (LOINC) reference data to LOINC codes. Doing so has revealed that LEAP would greatly benefit from a user interface to easily view relationships outside of an SQL environment. Also, we plan to build a dynamic SQL query so that mappings can be pivoted without hard-coding column names.

The LEAP framework transparently streamlines the process to harmonize data to various classification systems. This increases efficiency and visibility of the mapping process and ensures the integrity of the data being used, a challenge that existed during initial mapping attempts between GENIE and GDC. We believe that the LEAP framework could simplify future efforts of harmonizing classification elements. This transparent, easily shared process will reduce the operational and financial burden of informaticians in the future. Ultimately, we hope that this framework will be a helpful resource for future harmonization efforts as stakeholders strive toward a new taxonomy in the pursuit of precision medicine.

## DATA SHARING AND CODE AVAILABILITY

We share scripts to create and load the LEAP framework with all mapping data via GitHub at https://github.com/sbthomas1/GENIE-to-GDC. A workbook containing the data in the Synapse relational tables is available in the Data Supplement, as are files for both versions of OncoTree used in our mapping (oncotree_2017_06_21 and oncotree_2018_06_01).
